# Comprehensive Palliative Care Needs in Outpatients with Chronic Heart Failure: A Japanese Cross-Sectional Study

**DOI:** 10.1089/pmr.2021.0063

**Published:** 2022-04-18

**Authors:** Ryo Matsunuma, Kensuke Matsumoto, Takashi Yamaguchi, Akihiro Sakashita, Yoshiyuki Kizawa

**Affiliations:** ^1^ Department of Palliative Medicine, Kobe University Graduate School of Medicine, Kobe, Japan.; ^2^ Department of Palliative Care, Konan Medical Center, Kobe, Japan.; ^3^ Department of Cardiology, Kobe University Hospital, Kobe, Japan.; ^4^ Department of Palliative Medicine, Hyogo Brain and Heart Center, Himeji, Japan.

**Keywords:** chronic heart failure, Japan, outpatients, palliative care needs

## Abstract

**Background:**

The type and frequency of palliative care needs of chronic heart failure (CHF) patients have not been determined in Japan.

**Objectives:**

The aim of this study was to comprehensively assess the prevalence and characteristics of palliative care needs of CHF outpatients.

**Methods:**

Patients were recruited for this cross-sectional study from June 1 to August 31, 2020, at the Kobe University Hospital. An Integrated Palliative care Outcome Scale (IPOS) and an original questionnaire developed by multidisciplinary experts were answered once by patients themselves or with the assistance of their family.

**Results:**

A total of 101 patients (63 males and 38 females) were included. The most common distressing symptoms were dyspnea (29%; 95% confidence interval [CI] 21–39]), drowsiness (29%; 95% CI 21–39), poor mobility (25%; 95% CI 17–35), insomnia (25%; 95% CI 17–35), and anxiety (24%; 95% CI 17–35). Eighty percent (95% CI 70–87) of patients were willing to have an end-of-life (EOL) discussion. When we compared New York Heart Association class I/II with III/IV patients, the frequency of distressing symptoms was associated with the severity of the disease, but both groups exhibited a willingness for having an EOL discussion or knowing the future course of their diseases.

**Conclusions:**

Dyspnea, drowsiness, insomnia, and anxiety were frequent symptoms in CHF outpatients in Japan. Beyond distressing symptoms, most ambulatory heart failure patients have a need for EOL discussion, which was not associated with disease stage. Assessing comprehensive and multidimensional palliative care needs, including needs for EOL discussion, is advisable among outpatients with CHF.

## Introduction

Heart failure is recognized as a major and escalating public health problem in developed countries with aging populations.^1^ Worldwide, the prevalence of chronic heart failure (CHF) and its associated loss in health have been constantly increasing over the past several decades,^2^ and with an aging population, the number of CHF patients is expected to increase.

Previous studies reported that CHF patients have multidimensional unmet palliative care needs that span physical, psychological, social, spiritual, and informational aspects. Specifically, they include dyspnea, fatigue, edema, insomnia,^2–4^ and reduced exercise tolerance^5^ (physical); anxiety^4,6,7^ and depression^6,8,9^ (psychological); social isolation^7^ and lack of social support^9^ (social); maintaining hope, finding the meaning of their illness, the need for spiritual support,^3,10^ lack of hope, coping, faith, belief, and existential issues^11^ (spiritual); and the cause of their illness, the meaning of their symptoms, explanation of test results, the role of their medications, diagnosis and prognosis,^3^ and accurate information about their diagnosis^7^ (informational aspects). Thus, CHF patients require palliative care similar to cancer patients and should be cared for comprehensively.^11^

Indeed, the World Health Organization identified cardiovascular disease as requiring palliative care,^12^ and the European Association for Palliative Care recommends that palliative care be introduced to CHF patients early in their disease trajectory.^13–16^

In Japan, the importance of palliative care for heart failure is only beginning to be recognized. In 2017, the Japanese Society of Cardiology made recommendations for palliative care in its guidelines,^17^ and since 2018, a specialized palliative care team for end-stage heart failure has been covered by the national health insurance. However, the integration of heart failure care and palliative care is not sufficient, and even the type and frequency of palliative care needs of CHF patients have not been determined.

The purpose of this study was to comprehensively identify the prevalence and characteristics of palliative care needs in outpatients with CHF in Japan. The results of this study will serve as a basis for promoting the integration of cardiovascular and palliative care, which can address patients' needs in conjunction with treatment from an early stage of their illness.

## Methods

### Study design

This cross-sectional study recruited patients visiting a heart failure outpatient clinic at Kobe University Hospital, from June 1 to August 31, 2020. The heart failure clinic ran once a week with about 80 patients attending each month. After obtaining written consent from patients, the cardiologist asked participants to answer a questionnaire, by themselves or with the assistance of family, only once during the study period.

### Participants

This study included patients who (1) had been diagnosed with CHF by a cardiologist, (2) had regularly visited the heart failure clinic at Kobe University, (3) were aged 20 years or more, (4) were outpatients, and (5) had provided informed consent. “Regularly visited” was defined as patients who attended the outpatient clinic more than twice. Patients who (1) could not speak, read, or write Japanese, (2) were deemed to be inappropriate for investigation by a cardiologist due to a worsening general condition or severe depression or anxiety, (3) had cognitive impairment to such an extent that they were unable to respond to the questionnaire, or (4) had not regularly visited the heart failure clinic were excluded.

### Measurements

#### Questionnaire

To identify comprehensive palliative care needs in CHF patients, we considered that physical and psychological symptoms, spiritual and social distress, illness understanding, information needs, and daily practical needs should all be evaluated. The Integrated Palliative care Outcome Scale (IPOS) was a tool developed to measure palliative care needs. It consists of 17 items that represent the most important patient-reported concerns: 10 physical (pain, shortness of breath, lack of energy, nausea, vomiting, poor appetite, constipation, dry mouth, drowsiness, and poor mobility); 2 emotional (anxiety and depression); 1 spiritual; 2 communication (sharing patients' feelings and information needs); 1 family anxiety; and 1 practical issue.^18^ The IPOS was adopted to both cancer and noncancer patients, including CHF patients,^19,20^ and the Japanese version was validated.^21^

In addition to the original 17 items, we included three additional items on common physical symptoms in CHF (insomnia, edema, and abdominal distention).^22,23^ Each item was answered using a 5-point Likert scale: (0 = not at all; 1 = slightly; 2 = moderately; 3 = severely; 4 = overwhelmingly) for physical symptoms, (0 = not at all; 1 = occasionally; 2 = sometimes; 3 = most of the time; 4 = always) for psychological issues, and (0 = always; 1 = most of the time; 2 = sometimes; 3 = occasionally; 4 = not at all) for communication, spiritual, and practical issues.^24^ We used the seven-day, patient-reported version of IPOS in this study based on a previous study.^25^

Although we thought it essential to evaluate illness understanding, information needs, and daily practical needs for understanding the palliative care needs of CHF patients, there were no optimal questionnaires for these items validated in Japanese. Thus, we developed an original questionnaire to evaluate these items for this study. A palliative care physician (R.M.) drafted a preliminary version of the questionnaire according to the results of previous studies,^26–29^ which was then modified following discussion among the multidisciplinary specialists within our research team that included palliative care physicians, a cardiologist, palliative care nurses, a heart failure nurse specialist, and a palliative care pharmacy specialist. Questions are summarized below, with the full questionnaire provided in Supplementary Appendix SA1.

1. Daily practical needs: we asked participants to answer questions about the problems they face in living with heart failure, choosing from 19 items and allowing for multiple answers, adopted from problem lists used for distress screening in cancer patients.^27^
2. Illness understanding: we asked the following three questions about illness understanding. (1) Whether they knew the name of their disease using a binary question (yes or no). (2) To answer the type of treatment they were receiving in the cardiology clinic, choosing from the following five choices and allowing multiple answers (a, medication; b, pacemaker; c, implanted cardioverter defibrillator; d, left ventricular assist device; e, other.) (3) We asked about their understanding of the expected course of their disease using a 5-point Likert scale (1: definitely, 2: mostly, 3: unsure, 4: somewhat, 5: not at all) adopted from previous studies.^27,28^ Patients who selected “somewhat” or “not at all” were asked about their degree of willingness to know the expected course of their disease using the same 5-point Likert scale.
3. Experience around thinking about end-of-life (EOL) treatment and care, and preference for EOL discussion: we asked participants about their experience thinking about what treatment and care they would like to receive if they lost the decision-making capacity due to an advanced medical condition using a binary question (yes or no), adopted from a previous study.^29^ We also asked their degree of experience discussing what kind of treatment and care they would like to receive if they lost the decision-making capacity due to an advanced medical condition using a 5-point Likert scale (1: sufficiently discussed, 2: somewhat discussed, 3: neither discussed nor not discussed, 4: not really discussed, 5: not discussed at all).

Patients who selected “sufficiently discussed” or “somewhat discussed” were asked with whom it had been discussed, choosing from 12 variables and allowing for multiple answers. Patients were also asked about their willingness to discuss what kind of EOL treatment or care they would like to receive if they lost the decision-making capacity due to an advanced medical condition with others important to them and/or health care professionals using a 5-point Likert scale (1: very much, 2: a little, 3: neither, 4: not really, 5: not at all) adopted from a previous study.^28^

4. Preference for specific EOL treatment and care: we asked participants to answer what treatment and care would be acceptable or unacceptable if their condition deteriorated and they became incapacitated, choosing from 11 items and allowing for multiple answers. These 11 items were developed based on discussion among the authors.
5. Patient's values: we asked participants to answer what they would consider most important for them if their disease were to progress and they had only a limited time to live, choosing from 12 items and allowing for multiple answers. These 12 items were adopted from a previous study about components of a good death.^30^
6. Preference for receiving specialized palliative care: patients were asked if they would like to receive specialized palliative care using a binary question (yes or no).

The face validity of the questionnaire was confirmed through a pilot test on three conveniently sampled CHF patients.

#### Patient characteristics

Patient characteristics were extracted from electronic medical records, and included age, sex, comorbidities using the Charlson Comorbidity Index,^31^ and heart failure classification as per the American Heart Association/American College of Cardiology and the New York Heart Association (NYHA).^32^

#### Experience, current treatment, and devices related to CHF

The following data were extracted from medical records: history of percutaneous coronary intervention, radiofrequency catheter ablation for arrhythmia, surgical intervention for cardiovascular disease, and heart transplant; the presence of implanted pacemakers, implanted cardioverter defibrillators, cardiac resynchronization therapy defibrillators, and left ventricular assist devices; medications administered for heart failure; and patients who received home oxygen therapy and continuous positive airway pressure.

### Outcomes

The primary outcome of this study was to identify symptoms in CHF patients using IPOS. However, we considered that the identification of illness understanding and information needs were insufficient in IPOS. Therefore, as a secondary outcome, we designed an original questionnaire to capture palliative care needs not detected with IPOS.

### Analysis

A descriptive analysis was first performed to clarify the prevalence and characteristics of palliative care needs. In this study, palliative care needs were operationally defined as IPOS items when the following criteria were met: (1) physical symptoms of IPOS (if the patient answered 2 = moderately; 3 = severely; or 4 = overwhelmingly); (2) psychological issues of IPOS (if the patient answered 2 = sometimes; 3 = most of the time; or 4 = always); and (3) communication, spiritual, and practical issues of IPOS (if the patient answered 2 = sometimes; 3 = occasionally; or 4 = not at all). Patients were classified as having palliative care needs if they had “moderate,” “severe,” or “overwhelming” physical symptoms (IPOS), who “sometimes,” “most of the time,” or “always” had psychological issues, and who “sometimes,” “occasionally” or “not at all” felt at peace and shared their feelings with their family or friends.

To simplify interpretations, we converted the responses to several questions from a 5-point Likert scale to a binary response. The two questions on understanding and willingness to know the expected course of their disease were converted to 1: definitely, 2: mostly, versus others. The questions on the degree of experience for EOL discussion were converted to 1: sufficiently discussed, 2: somewhat discussed, versus others. Responses about the willingness to discuss what kind of EOL treatment or care they would like to receive were converted to 1: very much, 2: a little, versus others.

To determine the differences in palliative care needs based on disease severity, we stratified participants as either mild (NYHA class I and II) or moderate-to-severe (NYHA class III and IV) heart failure, and compared palliative care needs between the two groups. Continuous variables were analyzed using the Student's *t*-test, while categorical variables were analyzed using the chi-squared test. Statistical analyses were performed using SPSS software (version 26.0; IBM, Tokyo, Japan), where a two-tailed *p*-value <0.05 indicated statistical significance.

### Ethics

This study was conducted in accordance with the ethical standards of the Helsinki Declaration and the ethical guidelines for epidemiologic research of the Ministry of Health, Labour and Welfare in Japan. Oral and written informed consent was obtained from all participants by a cardiologist. The study was also approved by the Independent Ethics Committee of Kobe University Hospital (Approval No. B200046).

## Results

From June 1 to August 11, 2020, a total of 155 patients visited the heart failure clinic. Fifty-four patients met the exclusion criteria: did not regularly visit (*n* = 26), had cognitive impairment (*n* = 5), did not have CHF (*n* = 4), assessed by phone call to avoid severe acute respiratory syndrome coronavirus 2 infection (*n* = 2), could not read Japanese (*n* = 1), or considered inappropriate to participate as evaluated by the cardiologist (*n* = 5). Eleven patients refused to participate for various reasons (such as did not have enough time to reply, or did not want to reply). Ultimately, 101 patients (63 males and 38 females) were included.

Table 1 shows the characteristics of patients. Mean age was 65 ± 15 years, and 63% (95% confidence interval [CI] 52–71) were male. Patients with American Heart Association/American College of Cardiology stage C/D represented 46% (95% CI 36–55), and those with NYHA class III/IV represented 33% (95% CI 24–42). The most common etiology of heart failure was dilated cardiomyopathy (29%; 95% CI 21–38).

**Table 1.** Characteristics of Study Patients

Patient characteristics	*N* = 101
Age (mean ± SD)	65 ± 15
Male, *n* (%)	63 (63)
AHA/ACC stage, *n* (%)	
B	55 (55)
C	43 (43)
D	3 (3)
NYHA class, *n* (%)	
I	32 (32)
II	36 (36)
III	30 (30)
IV	3 (3)
Cause of CHF, *n* (%)	
Dilated cardiomyopathy	29 (29)
Valve disease	16 (16)
Hypertrophic cardiomyopathy	14 (14)
Ischemic heart disease	10 (10)
Cardiac sarcoidosis	13 (13)
Congenital heart disease	4 (4)
Cardiac amyloidosis	3 (3)
Others	15 (15)
Comorbidity, *n* (%)	
Chronic renal failure	22 (22)
Diabetes mellitus	19 (19)
Malignancy	19 (19)
Collagen disease	12 (12)
Cerebral vascular disease	10 (10)
Peripheral vascular disease	4 (4)
COPD	3 (3)
Dementia	1 (1)
Implemental devices, *n* (%)	
CRT	11 (11)
PM	9 (9)
ICD	8 (8)
LVAD	0
Medication, *n* (%)	
ACE	14 (14)
ARB	46 (46)
β-Blocker	88 (88)
MRA	34 (34)
Statin	31 (31)
Diuretic agents	51 (51)

ACC, American College of Cardiology; ACE, angiotensin-converting enzyme inhibitor; AHA, American Heart Association; ARB, angiotensin II receptor blockers; CHF, chronic heart failure; COPD, chronic obstructive pulmonary disease; CRT, cardiac resynchronization therapy; ICD, implantable cardioverter-defibrillators; LVAD, left ventricular assist device; MRA, mineralocorticoid receptor antagonist; NYHA, New York Heart Association; PM, pacemaker; SD, standard deviation.

### Symptoms

The IPOS results are described in Table 2. Eighty-five patients answered it by themselves, while 16 patients replied with family help. Dyspnea (29%; 95% CI 21–39) and drowsiness (29%; 95% CI 21–39) were the most common physical symptoms. Several patients suffered from psychological symptoms, including insomnia (25%; 95% CI 17–35), anxiety (25%; 95% CI 17–35), and depression (18%; 95% CI 11–27).

**Table 2.** Prevalence of Palliative Care Needs Using Integrated Palliative Care Outcome Scale and Comparison between New York Heart Association Class I/II and New York Heart Association Class III/IV Groups

IPOS variable	Total (*N* = 101), *n* (%)	NYHA I/II (*N* = 68), *n* (%)	NYHA III/IV (*N* = 33), *n* (%)	Class III/IV vs. Class I/II	
				OR (95% CI)	*p*
Pain	13 (13)	7 (11)	6 (18)	1.81 (0.56–5.91)	0.25
Dyspnea	29 (29)	16 (24)	13 (39)	2.07 (0.85–5.08)	0.11
Fatigue	**25 (26)**	**11 (17)**	**14 (42)**	**3.55 (1.38–9.16)**	**0.007**
Nausea	5 (5.3)	3 (5)	2 (6)	1.31 (0.21–8.28)	0.56
Vomiting	3 (3)	0	3 (9)	N/A	N/A
Appetite loss	**11 (11)**	**4 (6)**	**7 (21)**	**4.24 (1.14–15.7)**	**0.028**
Constipation	15 (15)	8 (12)	7 (22)	2.07 (0.68–6.31)	0.16
Dry mouth	19 (19)	11 (16)	8 (24)	1.66 (0.60–4.62)	0.33
Drowsiness	**29 (29)**	**13 (19)**	**16 (48)**	**3.91 (1.57–9.74)**	**0.003**
Poor mobility	**25 (25)**	**12 (18)**	**13 (39)**	**3.03 (1.19–7.74)**	**0.018**
Insomnia	23 (25)	18 (33)	5 (16)	0.46 (0.15–1.39)	0.16
Edema	10 (11)	6 (10)	4 (14)	1.47 (0.38–5.70)	0.41
Bowel distention	**14 (16)**	**6 (10)**	**8 (29)**	**3.6 (1.11–11.7)**	**0.031**
Patient anxiety	**24 (25)**	**11 (18)**	**13 (42)**	**3.55 (1.35–9.30)**	**0.008**
Family anxiety	**27 (28)**	**12 (14)**	**16 (48)**	**4.24 (1.68–10.7)**	**0.002**
Depression	17 (18)	9 (14)	8 (25)	2.04 (0.70–5.92)	0.19
Feeling at peace	15 (15)	7 (11)	8 (25)	2.76 (0.90–8.47)	0.066
Sharing feelings	24 (25)	14 (22)	11 (34)	1.87 (0.73–4.79)	0.19
Information	**24 (25)**	**11 (17)**	**14 (44)**	**3.68 (1.42–9.55)**	**0.006**
Practical issues	12 (13)	6 (9)	7 (23)	2.87 (0.87–9.42)	0.074

Bold variables mean significantly frequent in patients with NYHA IV.

CI, confidence interval; IPOS, Integrated Palliative care Outcome Scale; N/A, not analyzed; OR, odds ratio.

### Daily practical problems

The most frequent problem was with exercise (51%; 95% CI 39–63). Other common problems included eating (31%; 95% CI 21–43), going out (26%; 95% CI 17–38), household-related (20%; 95% CI 12–31), and finances (17%; 95% CI 9.5–28).

### Illness comprehension and EOL discussions

Ninety-six percent (95% CI 89–99) of patients understood the name of their disease, while 62% (95% CI 53–72) of the patients understood the expected course of their disease. Among the patients who did not understand the expected course of their disease (*n* = 34 [38%; 95% CI 29–49]), 79% (95% CI 62–90) of patients expressed a desire to know it. Forty percent (95% CI 30–50) of patients had experience thinking about what kind of treatment they would like to receive in potential future incapacity due to an advanced medical condition.

Forty-two percent (95% CI 32–52) of patients had had discussions on treatment and care if they lost the decision-making capacity at the end of their life. Eighty percent (95% CI 70–87) of patients answered that they had a willingness for such discussions with others important to them and/or health care professionals. These data are shown in Table 3.

**Table 3.** The Results of Illness Understanding, Knowledge of the Expected Course, Desire for Knowledge of the Expected Course, and Experience and Preference for End-of-Life Discussion in the Original Questionnaire

Items	No. of respondents (N = 101)	
	*n*	% (95% CI)
Knowing the name of their disease^a^	93	96 (90–98)
Understanding of the expected course of their disease^b^	56	62 (53–72)
Willingness to know the expected course of their disease^c^	27	79 (62–90)
Experience of thinking about EOL treatment and care^d^	36	40 (30–50)
Experience of discussing what kind of EOL treatment and care you would like to receive if you lost decision-making capacity due to an advanced medical condition^e^	37	42 (32–52)
Willingness to discuss what kind of treatment or care you would like to receive if you lost decision-making capacity due to an advanced medical condition with others^f^	72	80 (70–87)

^a^ Patients who answered “yes” on a binary question.

^b^ Patients who answered “1: definitely” or “2: mostly” on a 5-point Likert-like scale

^c^ Among patients who answered “4: somewhat” or “5: not at all” for the knowledge of the expected course of the disease (*n* = 34), patients who answered “1: definitely” or “2: mostly” on a 5-point Likert-like scale.

^d^ Patients who answered “yes” on a binary question.

^e^ Patients who answered “1: sufficiently discussed” or “2: somewhat discussed” on a 5-point Likert-like scale.

^f^ Patients who answered “1: very much” or “2: a little” on a 5-point Likert-like scale.

EOL, end of life.

### Acceptable and unacceptable EOL treatment and care

Chest compression (51%; 95% CI 38–63) and pacemaker (49%; 95% CI 36–62) were frequently selected as acceptable treatments, while percutaneous endoscopic gastrostomy (5.1%; 95% CI 1.2–14) and heart transplantation (8.5%; 95% CI 3.3–19) were less selected as acceptable.

### Patient values

The things that patients considered most important in their care if their disease were to progress and they had only a limited time to live were, in order of frequency, “being free from physical and psychological distress” (34%; 95% CI 25–45), “not being a burden to others” (32%; 95% CI 23–42), “spending enough time with one's family or friends” (24%; 95% CI 17–34), and “having no familial financial worries” (21%; 95% CI 14–31). Only one patient selected “receiving enough treatment” (1.1%; 95% CI −40 to 6.6).

Detailed results from the original questionnaire on daily practical needs, who the patients would like to have EOL discussion with, preference for specific EOL treatment and care, patient's values, and preference for receiving specialized palliative care are provided in Supplementary Appendix SA2.

### Comparison of needs between NYHA class I/II and III/IV patients

When we compared NYHA class I/II with NYHA class III/IV patients, attributes significantly more frequent in class III/IV patients included physical and psychological symptoms (Table 2). The physical symptoms included fatigue (I/II:III/IV = 17%:42%; odds ratio [OR] = 3.55; 95% CI 1.38–9.16; *p* = 0.007), appetite loss (I/II:III/IV = 6%:21%; OR = 4.24; 95% CI 1.14–15.7; *p* = 0.028), poor mobility (I/II:III/IV = 18%:39%; OR = 3.03; 95% CI 1.19–7.74; *p* = 0.018), abdominal distention (I/II:III/IV = 10%:29%; OR = 3.60; 95% CI 1.11–11.7; *p* = 0.031), and drowsiness (I/II:III/IV = 19%:48%; OR = 3.91; 95% CI 1.57–9.74; *p* = 0.003). Dyspnea tended to be more frequent in patients with NYHA III/IV, although this was not statistically significant (I/II:III/IV = 24%:39%; OR = 2.07; 95% CI 0.85–5.08; *p* = 0.007).

Psychological symptoms that were more frequent in NYHA class III/IV patients included patient anxiety (I/II:III/IV = 18%:42%; OR = 3.55; 95% CI 1.35–9.30; *p* = 0.008) and family anxiety (I/II:III/IV = 14%:48%; OR = 4.24; 95% CI 1.68–10.7; *p* = 0.002). There were no significant differences between patients with NYHA I/II and III/IV in regard to daily practical problems (Supplementary Appendix SA2).

As shown in Table 4, patients with NYHA III/IV had significantly more experience in thinking about EOL treatment and care if they lost their decision-making capacity. Moreover, patients who had experience in discussing what kind of EOL treatment and care they would like to receive if they lost their decision-making capacity due to an advanced medical condition were significantly more common in the NYHA III/IV group. Understanding of the expected course of their disease (I/II:III/IV = 69%:73%; OR = 1.21; 95% CI 0.45–3.22; *p* = 0.71), willingness to know the expected course of their disease (I/II:III/IV = 82%:75%; OR = 0.67; 95% CI 0.12–3.64; *p* = 0.48), and willingness to have EOL discussions with significant others and/or health care professionals (I/II:III/IV = 79%:82%; OR = 1.22; 95% CI 0.39–3.83; *p* = 0.73) were not significantly different between the two groups of patients.

**Table 4.** Comparison of Illness Understanding, Knowledge of the Expected Course, Desire for Knowledge of the Expected Course, and Experience and Preference for End-of-Life Discussion between New York Heart Association I/II and New York Heart Association III/IV Groups in the Original Questionnaire

Items	No. of respondents with NYHA class I/II (*N* = 68)		No. of respondents with NYHA class III/IV (*N* = 33)		Class III/IV vs. Class I/II	
	*n*	% (95% CI)	*n*	% (95% CI)	OR (95% CI)	*p*
Knowing the name of their disease^a^	63	97 (89–100)	30	94 (79–99)	2.10 (0.28–15.6)	0.40
Understanding of the expected course of their disease^b^	41	69 (57–80)	22	73 (55–86)	1.21 (0.45–3.22)	0.71
Willingness to know the expected course of their disease^c^	18	82 (61–93)	9	75 (46–92)	0.67 (0.12–3.64)	0.48
Experience of thinking about EOL treatment and care^d^	19	32 (21–44)	17	55 (38–71)	0.38 (0.16–0.93)	**0.032**
Experience of discussing what kind of EOL treatment and care you would like to receive if you lost decision-making capacity due to an advanced medical condition^e^	19	33 (22–46)	18	60 (42–75)	3.08 (1.24–7.68)	**0.014**
Willingness to discuss what kind of treatment or care you would like to receive if you lost decision-making capacity due to an advanced medical condition with others^f^	49	79 (67–87)	23	82 (64–93)	1.22 (0.39–3.83)	0.73

^a^ Patients who answered “yes” on a binary question.

^b^ Patients who answered “1: definitely” or “2: mostly” on a 5-point Likert-like scale.

^c^ Among the patients who answered “4: somewhat” or “5: not at all” for the knowledge of the expected course of the disease (*n* = 34), patients who answered “1: definitely” or “2: mostly” on a 5-point Likert-like scale.

^d^ Patients who answered “yes” on a binary question.

^e^ Patients who answered “1: sufficiently discussed” or “2: somewhat discussed” on a 5-point Likert-like scale.

^f^ Patients who answered “1: very much” or “2: a little” on a 5-point Likert-like scale.

## Discussion

To the best of our knowledge, this is the first study comprehensively evaluating palliative care needs in CHF outpatients in Japan. Our results revealed three major findings.

First, for physical symptoms, IPOS revealed that dyspnea, drowsiness, fatigue, and poor mobility were frequent symptoms in CHF outpatients in Japan. Roch et al. found that 74% and 68% of heart failure patients in Germany suffered from dyspnea and drowsiness, assessed by the IPOS, respectively,^33^ which is more frequent than we observed. The prevalence of psychological symptoms in our study was insomnia (25%; 95% CI 17–35), anxiety (25%; 95% CI 17–35), and depression (18%; 95% CI 11–27). These symptoms were again more frequent in Roch et al.'s study (anxiety: 56% vs. 25%; depression: 47% vs. 18%). In addition, clinical depression is prevalent among cancer patients with rates ranging between 13% and 40%.^34^ Thus, the prevalence of physical and psychological symptoms in our study was relatively low.

However, in Roch et al.'s study, 96% of patients were NYHA class III/IV, while only 33% of patients were NYHA class III/IV in our study. These differences in symptom frequencies may be due to Roch et al.'s study comprising a greater number of more severely affected patients. Therefore, to compare physical and psychiatric symptoms in patients with CHF in greater detail, it is necessary to study a population with a similar severity of illness.

Our second major finding was that 80% of patients had a willingness for EOL discussions with others important to them and/or health care professionals if they lost their decision-making capacity, and there was no difference between the patients with NYHA class III/IV and NYHA class I/II. Moreover, among patients who answered that they did not understand their expected course of the disease, 79% of them would like to have more information. Previous research found that CHF patients had information needs in relation to prognosis, treatment, cause of the disease, or trajectory of the disease.^27,35–37^

Caldwell et al. found that NYHA IV CHF patients desired discussion concerning resuscitation,^38^ however, there were no reports of information needs and desire for EOL discussion in patients with early stage of CHF, such as NYHA I/II. The European Association for Palliative Care recommends that palliative care should be provided using a symptoms and needs assessment-based approach, regardless of severity. Moreover, EOL discussion should be initiated at any stage of a person's life due to the unpredictable trajectory of CHF.^13^ In our study, we found that even if a patient's heart failure is early or mild in severity, they still had these information needs and desire for an EOL discussion. Therefore, it is advisable to screen information needs and desire for an EOL discussion regardless of the severity, and to initiate EOL discussions if appropriate.

Third, our results showed that half of the patients classified in NYHA class III/IV felt their family had anxiety about their illness. In a similar study, Roch et al. found that 79% of CHF patients felt family anxiety when assessed by the IPOS.^33^ Two cross-sectional studies found that 80% of patient caregivers had anxiety.^39,40^ This suggests that the family's palliative care needs may not be getting adequately addressed. The World Health Organization defines palliative care as an approach that improves the quality of life of patients and their families.^41^ Therefore, our results and previous studies suggest it may be advisable to include the palliative care needs of patients' families when palliative care needs are screened in CHF patients.

Our study showed that IPOS could identify the palliative care needs even in outpatients who were relatively stable. This demonstrates clinicians can detect the comprehensive palliative care needs of their patients using IPOS to provide them with appropriate palliative care.

### Limitations

There were several limitations in this study. First, since this study was based on a small number of patients from a single institution, there might be a selection bias and a problem in generalizability. This study investigated comprehensive palliative care needs, and the survey was conducted to the extent possible based on the number of outpatients at a heart failure clinic. As such, the number of participants was not accumulated to meet sufficient sample size calculations. Second, this study was a cross-sectional study. Therefore, it is possible that the survey captured the temporary needs at the time of the survey. Further research, such as using multiple or longitudinal surveys, may be necessary to more accurately capture palliative care needs.

Third, family anxiety was assessed from the perspective of the patient rather than the family. Therefore, this needs to be validated by the families to accurately determine their palliative care needs. Fourth, as our questioning of acceptable and unacceptable treatment did not provide detailed information, patients might have replied to these questions without fully understanding the treatments.

Furthermore, assessment of values and understanding of disease may not be sufficient because they were assessed using dichotomous questions. Additional study is needed to identify acceptable and unacceptable EOL care, values, and disease understanding in CHF patients. Finally, due to a lack of Japanese validated instruments to detect palliative care needs in CHF patients, we developed several questions based on literature review and specialist discussion, without a psychometric evaluation, such as reliability and validity. For a more accurate assessment, it would be advisable to conduct a qualitative study of the patient's and family's experience, develop a questionnaire based on the qualitative study, and conduct a psychometric validation before conducting the study.

## Conclusions

To the best of our knowledge, this is the first study to identify the palliative care needs among Japanese outpatients with CHF. This study revealed that dyspnea, drowsiness, fatigue, poor mobility, insomnia, anxiety, and depression were frequent symptoms in CHF outpatients in Japan. Although the prevalence of these physical and psychological symptoms was related to the severity of the disease, there was no significant difference between NYHA I/II and III/IV groups in relation to the willingness for information regarding the expected course of their disease and EOL discussions if they lost their decision-making capacity. A comprehensive and multidimensional assessment of needs, rather than severity or prognosis, should be advisable when palliative care intervention is being considered in CHF patients, regardless of disease severity.

## Supplementary Material

Supplemental data

Supplemental data

## Supplementary Material

Supplementary Appendix SA1

Supplementary Appendix SA2

## Abbreviations Used

ACC: American College of Cardiology
ACE: angiotensin-converting enzyme inhibitor
AHA: American Heart Association
ARB: angiotensin II receptor blockers
CHF: chronic heart failure
CI: confidence interval
COPD: chronic obstructive pulmonary disease
CRT: cardiac resynchronization therapy
EOL: end of life
ICD: implantable cardioverter-defibrillators
IPOS: Integrated Palliative care Outcome Scale
LVAD: left ventricular assist device
MRA: mineralocorticoid receptor antagonist
NYHA: New York Heart Association
OR: odds ratio
PM: pacemaker
SD: standard deviation

## References

[1] Groenewegen A, Rutten FH, Mosterd A, and Hoes AW: Epidemiology of heart failure. Eur J Heart Fail 2020;22:1342–1356. 32483830 10.1002/ejhf.1858 PMC7540043

[2] Park J, and Johantgen ME: A cross-cultural comparison of symptom reporting and symptom clusters in heart failure. J Transcult Nurs 2017;28:372–380. 27225884 10.1177/1043659616651673

[3] Namukwaya E, Grant L, Downing J, et al.: Improving care for people with heart failure in Uganda: Serial in-depth interviews with patients' and their health care professionals. BMC Res Notes 2017;10:1–13. 28545502 10.1186/s13104-017-2505-0 PMC5445313

[4] Davidson PM, Cockburn J, and Newton PJ: Unmet needs following hospitalization with heart failure: Implications for clinical assessment and program planning. J Cardiovasc Nurs 2008;23:541–546. 18953221 10.1097/01.JCN.0000338927.43469.35

[5] Ivany E, and While A: Understanding the palliative care needs of heart failure patients. Br J Community Nurs 2013;18:441–445. 24005488 10.12968/bjcn.2013.18.9.441

[6] O'Leary N, Murphy NF, O'Loughlin C, et al.: A comparative study of the palliative care needs of heart failure and cancer patients. Eur J Heart Fail 2009;11:406–412. 19196753 10.1093/eurjhf/hfp007

[7] Cagle JG, Bunting M, Kelemen A, et al.: Psychosocial needs and interventions for heart failure patients and families receiving palliative care support: A systematic review. Heart Fail Rev 2017;22:565–580. 28217818 10.1007/s10741-017-9596-5

[8] Fitzsimons D, Mullan D, Wilson JS, et al.: The challenge of patients' unmet palliative care needs in the final stages of chronic illness. Palliat Med 2007;21:313–322. 17656408 10.1177/0269216307077711

[9] Selman L, Beynon T, Higginson IJ, and Harding R: Psychological, social and spiritual distress at the end of life in heart failure patients. Curr Opin Support Palliat Care 2007;1:260–266. 18685372 10.1097/SPC.0b013e3282f283a3

[10] Park CL, and Sacco SJ: Heart failure patients' desires for spiritual care, perceived constraints, and unmet spiritual needs: Relations with well-being and health-related quality of life. Psychol Heal Med 2017;22:1011–1020. 10.1080/13548506.2016.1257813 27838924

[11] Ross L, and Austin J: Spiritual needs and spiritual support preferences of people with end-stage heart failure and their carers: Implications for nurse managers. J Nurs Manag 2015;23:87–95. 23859075 10.1111/jonm.12087

[12] World Palliative Care Alliance. 2014. Global Atlas of Palliative Care at the End of Life. www.who.int/cancer/publications/palliative-care-atlas/en (Last accessed July 30, 2021).

[13] Sobanski PZ, Alt-Epping B, Currow DC, et al.: Palliative care for people living with heart failure: European Association for Palliative Care Task Force expert position statement. Cardiovasc Res 2020;116:12–27. 31386104 10.1093/cvr/cvz200

[14] Evangelista LS, Liao S, Motie M, et al.: On-going palliative care enhances perceived control and patient activation and reduces symptom distress in patients with symptomatic heart failure: A pilot study. Eur J Cardiovasc Nurs 2014;13:116–123. 24443421 10.1177/1474515114520766 PMC4455924

[15] Diop MS, Rudolph JL, Zimmerman KM, et al.: Palliative care interventions for patients with heart failure: A systematic review and meta-analysis. J Palliat Med 2017;20:84–92. 27912043 10.1089/jpm.2016.0330 PMC5177994

[16] Akyar I, Dionne-Odom JN, and Bakitas MA: Using patients and their caregivers feedback to develop ENABLE CHF-PC: An early palliative care intervention for advanced heart failure. J Palliat Care 2019;34:103–110. 29952216 10.1177/0825859718785231

[17] Tsutsui H, Isobe M, Ito H, et al.: JCS 2017/JHFS 2017 guideline on diagnosis and treatment of acute and chronic heart failure—Digest version. Circ J 2019;83:2084–2184. 31511439 10.1253/circj.CJ-19-0342

[18] Higginson IJ, Simon ST, Benalia H, et al.: Which questions of two commonly used multidimensional palliative care patient reported outcome measures are most useful? Results from the European and African PRISMA survey. BMJ Support Palliat Care 2012;2:36–42. 10.1136/bmjspcare-2011-000061 24653497

[19] Collins ES, Witt J, Bausewein C, et al.: A systematic review of the use of the Palliative Care Outcome Scale and the Support Team Assessment Schedule in palliative care. J Pain Symptom Manage 2015;50:842–853.e19. 10.1016/j.jpainsymman.2015.07.015 26335764

[20] Fendler TJ, Swetz KM, and Allen LA: Team-based palliative and end-of-life care for heart failure. Heart Fail Clin 2015;11:479–498. 26142643 10.1016/j.hfc.2015.03.010 PMC4491937

[21] Sakurai H, Miyashita M, Imai K, et al.: Validation of the Integrated Palliative care Outcome Scale (IPOS)—Japanese Version. Jpn J Clin Oncol 2019;49:257–262. 30668720 10.1093/jjco/hyy203

[22] Redeker NS, Jeon S, Muench U, et al.: Insomnia symptoms and daytime function in stable heart failure. Sleep 2010;33:1210–1216. 20857868 10.1093/sleep/33.9.1210 PMC2938862

[23] Mant J, Doust J, Roalfe A, et al.: Systematic review and individual patient data meta-analysis of diagnosis of heart failure, with modelling of implications of different diagnostic strategies in primary care. Health Technol Assess (Rockv) 2009;13:1–207. 10.3310/hta13320 19586584

[24] Beck I, Olsson Möller U, Malmström M, et al.: Translation and cultural adaptation of the Integrated Palliative care Outcome Scale including cognitive interviewing with patients and staff. BMC Palliat Care 2017;16:1–10. 28893215 10.1186/s12904-017-0232-x PMC5594532

[25] Oriani A, Guo P, Gadoud A, et al.: What are the main symptoms and concerns reported by patients with advanced chronic heart failure?—A secondary analysis of the palliative care outcome scale (POS) and integrated palliative care outcome scale (IPOS). Ann Palliat Med 2019;8:775–780. 31594366 10.21037/apm.2019.08.10

[26] NCCN. NCCN distress thermometer and problem list for patients. https://www.nccn.org/docs/default-source/patient-resources/nccn_distress_thermometer.pdf?sfvrsn=ef1df1a2_4 (Last accessed July 30, 2021).

[27] Harding R, Selman L, Beynon T, et al.: Meeting the communication and information needs of chronic heart failure patients. J Pain Symptom Manage 2008;36:149–156. 18599259 10.1016/j.jpainsymman.2007.09.012

[28] Malhotra C, Sim D, Jaufeerally F, and Finkelstein EA: Associations between understanding of current treatment intent, communication with healthcare providers, preferences for invasive life-sustaining interventions and decisional conflict: Results from a survey of patients with advanced heart failure in Singa. BMJ Open 2018;8:e021688. 10.1136/bmjopen-2018-021688 PMC6150135 30232107

[29] Young KA, Redfield MM, Strand JJ, and Dunlay SM: End-of-life discussions in patients with heart failure. J Card Fail 2017;23:821–825. 28842378 10.1016/j.cardfail.2017.08.451 PMC5671349

[30] Miyashita M, Kawakami S, Kato D, et al.: The importance of good death components among cancer patients, the general population, oncologists, and oncology nurses in Japan: Patients prefer “fighting against cancer.” Support Care Cancer 2015;23:103–110. 10.1007/s00520-014-2323-z 24996829

[31] Charlson ME, Charlson RE, Peterson JC, et al.: The Charlson comorbidity index is adapted to predict costs of chronic disease in primary care patients. J Clin Epidemiol 2008;61:1234–1240. 18619805 10.1016/j.jclinepi.2008.01.006

[32] Yancy CW, Jessup M, Bozkurt B, et al.: 2013 ACCF/AHA guideline for the management of heart failure: Executive summary: A report of the American College of Cardiology foundation/American Heart Association task force on practice guidelines. J Am Coll Cardiol 2013;62:1495–1539. 10.1016/j.jacc.2013.05.019 23747642

[33] Roch C, Palzer J, Zetzl T, et al.: Utility of the integrated palliative care outcome scale (IPOS): A cross-sectional study in hospitalised patients with heart failure. Eur J Cardiovasc Nurs 2020;19:702–710. 32370552 10.1177/1474515120919386

[34] Sellick SM, and Crooks DL: Depression and cancer: An appraisal of the literature for prevalence, detection, and practice guideline development for psychological interventions. Psychooncology 1999;8:315–333. 10474850 10.1002/(SICI)1099-1611(199907/08)8:4<315::AID-PON391>3.0.CO;2-G

[35] Kitakata H, Kohno T, Kohsaka S, et al.: Prognostic understanding and preference for the communication process with physicians in hospitalized heart failure patients. J Card Fail 2021;27:318–326. 33171293 10.1016/j.cardfail.2020.10.009

[36] Andersson L, and Nordgren L: Heart failure patients' perceptions of received and wanted information: A cross-sectional study. Clin Nurs Res 2019;28:340–355. 29986617 10.1177/1054773818787196

[37] Kimani KN, Murray SA, and Grant L: Multidimensional needs of patients living and dying with heart failure in Kenya: A serial interview study. BMC Palliat Care 2018;17:1–8. 10.1186/s12904-018-0284-6 PMC5816535 29454383

[38] Caldwell PH, Arthur HM, and Demers C: Preferences of patients with heart failure for prognosis communication. Can J Cardiol 2007;23:791–796. 17703257 10.1016/s0828-282x(07)70829-2 PMC2651384

[39] Dirikkan F, Arabacı LB, and Mutlu E: The caregiver burden and the psychosocial adjustment of caregivers of cardiac failure patients. Turk Kardiyol Dern Ars 2018;46:692–701. 30516527 10.5543/tkda.2018.10.5543/tkda.2018.69057

[40] Hooley PJD, Butler G, and Howlett JG: The relationship of quality of life, depression, and caregiver burden in outpatients with congestive heart failure. Congest Heart Fail 2005;11:303–310. 16330905 10.1111/j.1527-5299.2005.03620.x

[41] World Health Organization: World Health Organization (WHO) Definition of Palliative Care. Cancer WHO Defin Palliat Care. 2002.

