# Supplementary material for: Comprehensive Palliative Care Needs in Outpatients with Chronic Heart Failure: A Japanese Cross-Sectional Study
Source: Palliat Med Rep. 2022 Apr 18;3(1):65–74. doi: 10.1089/pmr.2021.0063 (PMC9081025; doi:10.1089/pmr.2021.0063)
Supplement: Supplemental data [file Suppl_AppendixSA1.docx]

**Appendix 1: Questionnaire**

Please answer the following questions as a reference for medical care.

- 1. Do you have any problems living with CHF now?

Please check all applicable of these 19 items

| 1. Eating | 8. Finance | 15. Religion |
| --- | --- | --- |
| 1. Exercise | 9. Moving around in the house | 16. Appearance |
| 1. Going out | 10. Work/school | 17. Bathing/dressing |
| 1. Defecation and urination | 11. Treatment decisions | 18. Sexual |
| 1. Household | 12. Dealing with children | 19. Others |
| 1. Child care | 13. Dealing with partner | （　　　　　　　　　　　） |
| 1. Housing | 14. Family issues |  |

**2-1** Do you know what disease you are visiting a cardiology clinic for?

a. Yes b. No

**2-2** This question is for those who chose "a. Yes" in the question above. Please write the name of the disease.

⇒[　　　　　　　　　　　　　　　　　　　　]

**3．** What kind of treatment do you receive for diseases that you are currently visiting the cardiology clinic for?

Please choose all applicable of 5 items below.

a. Medication b. Pacemaker c. Implanted cardioverter defibrillator (ICD)

d. Left ventricular assist devices (LVADs) e. Others ( )

**4-1** Do you know the expected course of your disease?

1. Definitely b. Mostly c. Unsure d. Somewhat e. Not at all

The state of understanding the future course of the disease means, for example, that you have the following outlook.

For example

Example 1) I am currently being treated with medication, but my disease will improve in a couple of years and I will be able to stop the medication.

Example 2) I need to continue with my current treatment, but my condition will remain unchanged.

Example 3) My current disease is difficult to cure, and it will gradually progress and get worse.

**4-2**  For patients who selected ‘somewhat’ or ‘not at all’ in question 4-1, do you want to know the expected course of your disease?

a. Definitely b. Mostly c. Unsure d. Somewhat e. Not at all

**5-1** As the heart disease progresses, you may not be able to think deeply on your own or communicate your thoughts and feelings to others.

Have you thought about what treatment and care you would like to receive if you lost decision-making capacity due to an advanced medical condition?

a. Yes b. No.

**5-2** If your condition deteriorated due to disease progression you may not be able to think deeply on your own or communicate your thoughts and feelings with your family or friends.

Have you discussed what treatment and care you would like to receive if you lost decision-making capacity due to an advanced medical condition?

a. sufficiently discussed b. somewhat discussed c. neither discussed nor not discussed

d. not really discussed　e. Not discussed at all

**5-3** For patients who selected a. sufficiently discussed b. somewhat discussed in question 5-2, with whom was this discussed?

Please select all applicable of these 13 items.

| 1. Spouse | 4. Parents | 7. Friends | 10. Care Manager | 13. Others |
| --- | --- | --- | --- | --- |
| 1. Children | 5. Grandparents | 8. Primary doctor | 11. Home care worker | （　　　　　） |
| 1. Brothers or sisters | 6. Grandchild | 9. Nurse | 12. Social Worker |  |

**6.** If your condition deteriorated due to disease progression you may not be able to think deeply on your own or communicate your thoughts and feelings with your family or friends.

Would you like to discuss what kind of treatment or care you would like to receive with others (e.g., a trusted family member, friend or healthcare professional)?

1. Very much b. A little c. Neither d. Not really e. Not at all
2. If your condition deteriorated due to disease progression you may not be able to think deeply on your own or communicate your thoughts and feelings with your family or friends.

Is there any treatment or care that you would be willing to receive?

Please select all applicable of 11 items

| 1. Chest compression | 5. Ventricular assist devices | 9. Administration of vasopressors |
| --- | --- | --- |
| 1. Mechanical ventilation | 6. Heart Transplant | 10. Admission to intensive care unit |
| 1. Hemodialysis | 7. Feeding through gastrostomy | 11. Others |
| 1. Pacemaker | 8. Nutrition by drip | （　　　　　　　　　　　） |

1. If your condition deteriorated due to disease progression you may not be able to think deeply on your own or communicate your thoughts and feelings with your family or friends.

Is there any treatment or care that you would **NOT** be willing to receive?

Please select all applicable of 11 items

| 1. Chest compression | 5. Ventricular assist devices | 9. Administration of vasopressors |
| --- | --- | --- |
| 1. Mechanical ventilation | 6. Heart Transplant | 10. Admission to intensive care unit |
| 1. Hemodialysis | 7. Feeding through gastrosomy | 11. Others |
| 1. Pacemaker | 8. Nutrition by drip | （　　　　　　　　　　　） |

1. If your disease were to progress and you had only a limited time to live, what would be the most important thing for you? Please select one of the 12 items.

| 1. Spending enough time with one’s family or friends | 1. Maintaining hope and pleasure |
| --- | --- |
| 1. Working or social activities | 1. Doing what one wants to do |
| 1. Being independent in daily activities | 1. Being able to stay at one’s favorite place |
| 1. Receiving enough treatment | 1. Having no own financial worries |
| 1. Not being a burden to others | 1. Having no familial financial worries |
| 1. Being free from physical and psychological distress | 1. Others ( ) |

1. Would you like to receive specialized palliative care?

a. Yes b. No

- Palliative care is specialized medical care for people living with a serious illness and their caregivers. This type of care is focused on providing relief from the physical, psychological, social and spiritual symptoms. It is provided by all medical professionals as part of daily medical care.
- "Specialized palliative care" is palliative care provided by specialists. In this institution, specialized palliative care is provided by ‘palliative care team’, an organized specially-trained team of doctors, nurses, pharmacists, psychologists, nutritionists, physiotherapists, etc.

Thank you for your answer.
