# Supplementary material for: Comprehensive Palliative Care Needs in Outpatients with Chronic Heart Failure: A Japanese Cross-Sectional Study
Source: Palliat Med Rep. 2022 Apr 18;3(1):65–74. doi: 10.1089/pmr.2021.0063 (PMC9081025; doi:10.1089/pmr.2021.0063)
Supplement: Supplemental data [file Suppl_AppendixSA2.docx]

**Appendix 2. The results of daily practical needs, who patients had end-of-life discussions with, preference for specific end-of-life treatment and care, patients’ values, and preference for receiving specialized palliative care in the original questionnaire**

| **Questions** |  |  |
| --- | --- | --- |
|  | **Answer** | **Number of respondents,**  **n (%, 95% CI)** |
| Do you have any problems living with CHF now? | | 65^a^ |
|  | Eating | 20 (30, 21-43) |
|  | Exercise | 33 (51, 39-63) |
|  | Going out | 17 (26, 17-38) |
|  | Defecation and urination | 4 (6.2, 2.0-15) |
|  | Household | 13 (20, 12-31) |
|  | Child care | 1 (1.5, -0.5-9.0) |
|  | Housing | 3 (4.6, 1.1-13) |
|  | Finance | 11 (17, 9.5-28) |
|  | Moving around in the house | 3 (4.6, 1.1-13) |
|  | Work/school | 8 (12, 6.1-23) |
|  | Treatment decisions | 6 (9.2, 4.0-19) |
|  | Dealing with children | 1 (1.5, -0.5-9.0) |
|  | Dealing with partner | 6 (9.2, 4.0-19) |
|  | Family issues | 5 (7.7, 2.9-17) |
|  | Religion | 1 (1.5, -0.5-9.0) |
|  | Appearance | 1 (1.5, -0.5-9.0) |
|  | Bathing/dressing | 5 (7.7, 2.9-17) |
|  | Sexual | 2 (3.1, 0.22-11) |
| If patients had experienced end-of-life discussions, with whom had patients had discussions with? | | 48^a^ |
|  | Spouse | 24 (50, 36-64) |
|  | Parents | 3 (6.3, 1.5-17) |
|  | Children | 26 (54, 40-67) |
|  | Brothers or sisters | 6 (13, 5.5-25) |
|  | Parents | 3 (6.3, 1.5-17) |
|  | Grandparents | 0 |
|  | Grandchild | 2 (4.2, 0.37-15) |
|  | Friends | 4 (8.3, 2.8-20) |
|  | Primary doctor | 11 (23, 13-37) |
|  | Nurse | 2 (4.2, 0.37-15) |
|  | Care manager | 1 (2.1, –0.64-12) |
|  | Home care worker | 1 (2.1, –0.64-12) |
|  | Social worker | 0 |
|  | Others | 1 (2.1, –0.64-12) |
|  |  |  |
| *If your condition deteriorated due to disease progression you may not be able to think deeply on your own or communicate your thoughts and feelings with your family or friends.* | | |
| Is there any treatment or care that you would be willing to receive? | | 59^a^ |
|  | Chest compression | 30 (51, 38-63) |
|  | Mechanical ventilation | 14 (24, 15-36) |
|  | Hemodialysis | 9 (15, 8.0-27) |
|  | Pacemaker | 29 (49, 37-62) |
|  | Ventricular assist devices | 8 (14, 6.8-25) |
|  | Heart Transplant | 5 (8.5, 3.3-19) |
|  | Feeding through gastrostomy | 3 (5.1, 1.2-14) |
|  | Nutrition by drip | 8 (14, 6.8-25) |
|  | Administration of vasopressors | 12 (20, 12-32) |
|  | Admission to intensive care unit | 17 (29,19-41) |
| Is there any treatment or care that you would **NOT** be willing to receive? | | 65^a^ |
|  | Chest compression | 14 (22, 13-33) |
|  | Mechanical ventilation | 24 (37,26-49) |
|  | Hemodialysis | 33 (51, 39-63) |
|  | Pacemaker | 16 (25, 16-36) |
|  | Ventricular assist devices | 31 (48, 36-60) |
|  | Heart Transplant | 43 (66, 54-77) |
|  | Feeding through gastrostomy | 41 (63, 51-74) |
|  | Nutrition by drip | 18 (28, 18-40) |
|  | Administration of vasopressors | 8 (12, 6.1-23) |
|  | Admission to intensive care unit | 19 (29, 20-41) |
|  | Others |  |
| If your disease were to progress and you had only a limited time to live, what would be the most important thing for you? | | 90^a^ |
|  | Spending enough time with one’s family or friends | 22 (24, 17-34) |
|  | Working or social activities | 5 (5.6, 2.1-13) |
|  | Being independent in daily activities | 13 (14, 8.5-23) |
|  | Receiving enough treatment | 1 (1.1, –0.40-6.6) |
|  | Not being a burden to others | 29 (32, 23-42) |
|  | Being free from physical and psychological distress | 31 (34, 25-45) |
|  | Maintaining hope and pleasure | 10 (11, 6.0-19) |
|  | Doing what one’s want to do | 12 (13, 7.6-22) |
|  | Being able to stay at one’s favorite place | 5 (5.5, 2.1-13) |
|  | Having no own financial worries | 7 (7.8, 3.6-15) |
|  | Having no familial financial worries | 19 (21, 14-31) |
|  | Others | 0 |
| Would you like to receive specialized palliative care? | | 96^a^ |
|  | Yes | 48 (50, 40-60) |

^a^ Total number of respondents, n

CI: confidence interval, NYHA: New York Heart Association, OR: odds ratio.
